# Structural equation modeling for decomposing rank-dependent indicators of socioeconomic inequality of health: an empirical study

**DOI:** 10.1186/s13561-016-0134-2

**Published:** 2016-12-07

**Authors:** Roselinde Kessels, Guido Erreygers

**Affiliations:** 1 Department of Economics, University of Antwerp and Flemish Research Foundation (FWO), City Campus, Prinsstraat 13, Antwerp, 2000 Belgium; 2 Department of Economics, University of Antwerp and Centre for Health Policy, University of Melbourne, City Campus, Prinsstraat 13, Antwerp, 2000 Belgium

**Keywords:** Inequality measurement, Generalized health Concentration Index, Decomposition methods, Structural Equation Modeling, C36, D63, I00

## Abstract

We present a flexible structural equation modeling (SEM) framework for the regression-based decomposition of rank-dependent indicators of socioeconomic inequality of health and compare it with simple ordinary least squares (OLS) regression. The SEM framework forms the basis for a proper use of the most prominent one- and two-dimensional decompositions and provides an argument for using the bivariate multiple regression model for two-dimensional decomposition. Within the SEM framework, the two-dimensional decomposition integrates the feedback mechanism between health and socioeconomic status and allows for different sets of determinants of these variables. We illustrate the SEM approach and its outperformance of OLS using data from the 2011 Ethiopian Demographic and Health Survey.

## Background

The dominant approach to the measurement of socioeconomic inequality of health consists of using rank-dependent indicators. They are called rank-dependent because they can be expressed as weighted averages of individual health levels, with the weights determined by the ranks of individuals in the socioeconomic distribution. Indices of this type allow us to find out whether there is pro-rich or pro-poor bias in the health distribution: positive values indicate that people who are relatively well-off in socioeconomic terms tend to have better health than those who are less well-off, and negative values the opposite. The standard health Concentration Index [24] is undoubtedly the most popular rank-dependent index. There is now also a growing literature on the decomposition of the Concentration Index using various econometric techniques (see, e.g., [1, 10, 21, 25]). An overview of recent contributions on the measurement and decomposition of socioeconomic inequality of health can be found in [10, 19, 20].

Compared to indicators of income inequality or health inequality, which measure the degree of inequality within a given univariate distribution of income or health, indicators of socioeconomic inequality of health are bivariate in nature because they measure the degree of *correlation* between health and socioeconomic status. To explain the degree of correlation between these two variables rather than the degree of inequality in one variable, Erreygers and Kessels [4] proposed a set of two-dimensional decompositions that investigate both variables simultaneously. The most salient of these decompositions is based on the bivariate multiple regression model that explains health and socioeconomic status simultaneously. This decomposition captures not only the direct contributions of the explanatory variables in the regressions, but also their combined or correlated contributions.

However, two criticisms may be made of the two-dimensional decomposition analysis based on the bivariate multiple regression model. The first is that the bivariate multiple regression model uses the same set of variables to explain both health and socioeconomic status, which may not be the most appropriate assumption given that the determinants of health and socioeconomic status need not be the same. Related to this, the second criticism is that socioeconomic status is not included as an explanatory variable in the regression of health, and health not included as an explanatory variable in the regression of socioeconomic status. The existence of a reciprocal relationship should be examined since health is potentially both a cause and a consequence of socioeconomic status [7, 16]. In the literature on the decomposition of socioeconomic inequality of health, several empirical studies (see, e.g., [2, 11, 13, 17, 18, 25]) have investigated the impact of socioeconomic status on health, reporting evidence that socioeconomic status is an important determinant of health. Gerdtham et al. [7] argue, however, that an overall consensus about the causal impact of socioeconomic status on health has not yet been reached.

The main objective of Erreygers and Kessels [4] was to compare the two-dimensional decomposition to the one-dimensional decompositions that are based on regressions of only one of the two variables under consideration. Therefore, they used the same set of explanatory variables in all regressions, which are all estimated using ordinary least squares (OLS). Moreover, for the one-dimensional decompositions, they argued that including either of the variables as an explanatory variable in the single regressions distorts the explanation of the correlation between health and socioeconomic status. It is then as if the variable in question were treated both as a dependent and as an independent variable. As a result, for the two-dimensional decomposition, a bivariate multiple regression modeling framework was chosen which includes neither health nor socioeconomic status as an explanatory variable.

To bridge the gap between empirical observations and modeling practice, we propose a flexible modeling approach for the decomposition of socioeconomic inequality of health that makes use of a structural or simultaneous equation model (SEM). The model allows for different sets of determinants of health and socioeconomic status as well as for the inclusion of socioeconomic status as an explanatory variable in the regression of health and health as an explanatory variable in the regression of socioeconomic status. The model produces consistent estimates of the regression coefficients using a two-step generalized method of moments (GMM) estimation procedure that includes instrumental variables. Although such a modeling approach has been hinted at before ([25]: 214, n.12) and commented upon for its data requirements which may be demanding [10], this paper is the first to adopt a SEM in a simple manner using real data.

The outline of the remainder of the paper is as follows. First, we review the Generalized health Concentration Index and the various concepts it embraces. Next, we provide an overview of the most important one- and two-dimensional decompositions, based on the OLS regression approach. After that, we present a flexible SEM approach for regression-based decomposition analysis and show how it fits with the existing decompositions. By means of an empirical analysis of child malnutrition in Ethiopia, we then illustrate the proper use of the one- and two-dimensional decompositions within the OLS and SEM regression framework. Finally, we summarize the paper and discuss the main outcomes.

## Methods

### Generalized health Concentration Index

We consider a population of *n* individuals for which the health level of individual *i*, denoted as *h*_*i*_, is either a ratio-scale variable which takes non-negative values only, or a cardinal variable with a finite lower bound. The average health level in the population is equal to $\mu _{h}=\frac {1}{n}\sum _{i=1}^{n}h_{i}$.

Erreygers and Van Ourti [6] pointed out that the use of the health Concentration Index is pertinent when we are dealing with a ratio-scale health variable which is unbounded, i.e. which does not have a finite upper bound. However, when we are dealing with a variable which has a finite upper bound, a modified version is called for. For this situation, Wagstaff [23] and Erreygers [3] each proposed a variant of the Generalized Concentration Index.

All these indices belong to the family of rank-dependent indices: they can be expressed as weighted sums of health levels with the weights determined by socioeconomic ranks. The socioeconomic rank of individual *i* is determined by his/her position according to the variable chosen to measure socioeconomic well-being, e.g. income. Let the value of this variable for individual *i* be *y*_*i*_. Then the natural number *r*_*i*_(*y*), or more simply *r*_*i*_, measures the position of individual *i* in the rank-order according to variable *y*, with the rank *r*_*i*_=1 assigned to the person who is least well-off, and the rank *r*_*i*_=*n* assigned to the person who is most well-off. In the case of ties, we assign to every individual of the tied group the average rank of the group. Over the population as a whole the average rank is $\mu _{r}=\frac {n+1}{2}$. The fractional rank *f*_*i*_ is defined as $f_{i}\equiv \frac {1}{n}\left (r_{i}-\frac {1}{2}\right) $, and varies between $\frac {1}{2n}$ and $1-\frac {1}{2n}$. The average fractional rank is $\mu _{f}=\frac {1}{2}$. Finally, the deviation of the fractional rank of individual *i* from the average fractional rank, denoted as *d*_*i*_≡*f*_*i*_−*μ*_*f*_, has an average of *μ*_*d*_=0.

The Generalized health Concentration Index *GC* is defined as:

1 $$ GC=\frac{2}{n}\sum\limits_{i=1}^{n}h_{i}d_{i}  $$

The standard health Concentration Index, *C*, as well as the indices introduced by Wagstaff [23], *W*, and by Erreygers [3], *E*, can be expressed as simple functions of *GC*:

2 $$\begin{array}{*{20}l} C&=\frac{1}{\mu_{h}}GC \end{array} $$

3 $$\begin{array}{*{20}l}  W&=\frac{b_{h}-a_{h}}{(b_{h}-\mu_{h})(\mu_{h}-a_{h})}GC \end{array} $$

4 $$\begin{array}{*{20}l} E&=\frac{4}{b_{h}-a_{h}}GC  \end{array} $$

where *a*_*h*_ and *b*_*h*_ stand for the lower and upper bounds of the health variable. Strictly speaking, the decompositions discussed in this paper are only applicable to *GC* and *E*. As argued by Heckley et al. [10], only these two indices comply with the assumption of weighting function ignorability required for proper decomposition analysis. This assumption states that the predictors of health do not influence the weighting function that is specific to each form of rank-dependent index and equal to the multiplier of *GC* in formulas (2)–(4). The two indices *GC* and *E* are characterized by a constant weighting function, which equals 1 for *GC* and $\frac {4}{b_{h}-a_{h}}$ for *E*. The other indices *C* and *W* have weighting functions that are functions of the mean health, and therefore also functions of the predictors of health. They thus violate the assumption of weighting function ignorability. In the remainder of the paper, we will concentrate on the decomposition of *GC* which is the basic index satisfying weighting function ignorability.

We can rewrite the formula for *GC* using a well-known relationship between the rank-dependent indices and the covariance. Since $Cov(h,d)=\frac {1}{n}\sum _{i=1}^{n}h_{i}d_{i}-\mu _{h}\mu _{d}$ and *μ*_*d*_=0, the value for *GC* can also be computed as:

5 $$ GC=2Cov(h,d)  $$

Erreygers and Kessels [4] used both (1) and (5) to generate decompositions of the Generalized Concentration Index. Some of these decompositions have a constant term. Because it is problematic to give a meaningful interpretation to the constant term, the most attractive decompositions are those without a constant term. In the next section, we review these decompositions, two of which are one-dimensional and one that is two-dimensional. In the one-dimensional decompositions, either the health variable or the fractional rank deviation variable is subject to a regression, whereas in the two-dimensional decomposition, both variables are subject to a regression. The regression approach used is simply OLS.

### One- and two-dimensional decompositions using OLS regression

#### The health-oriented decomposition

The health-oriented decomposition, introduced by Wagstaff et al. [25], has been the first and most well-known regression-based decomposition. It starts from the linear regression model describing the relationship between the health variable *h* and a number of explanatory variables *x*_1_,*x*_2_,...,*x*_*k*_:

6 $$ h_{i}=\beta_{0}+\beta_{1}x_{1,i}+\beta_{2}x_{2,i}+...+\beta_{k}x_{k,i}+\varepsilon_{i}  $$

where *ε*_*i*_ is an error term. Substituting the right-hand side of this model for *h*_*i*_ in the ‘product definition’ of the *GC* in (1) and working out the result, we obtain the health-oriented decomposition, henceforth referred to as decomposition (I):

7 $$ GC=2\sum\limits_{j=1}^{k}\beta_{j}Cov(x_{j},d)+2Cov(\varepsilon,d)   $$

This decomposition has a deterministic component consisting of a sum of *k* contributions, one for each explanatory variable, and a residual component.

As argued by Erreygers and Kessels [4] and Heckley et al. [10], it is misleading to include the fractional rank deviation variable *d* in the OLS regression for *h* in decomposition (I), or any proxy variable strongly correlated with *d* such as income or consumption. In that case, the residual component will be zero, or close to zero, suggesting that we have explained all or most of the variation in the Generalized Concentration Index. This result is, however, merely an artefact from the OLS regression-based approach of decomposition (I). Consider, for example, the simple case where the variable *d* is the only explanatory variable of *h*, i.e. *x*_1_=*d*. Since the OLS estimate of *β*_1_ is then equal to *C* *o* *v*(*h*,*d*)/*V* *a* *r*(*d*), it follows that the deterministic component of decomposition (I) is identical to *GC* and therefore the residual component equal to zero. However, in this case, we have explained nothing at all. We are just treating the fractional rank deviation variable *d* both as a dependent and as an independent variable. In other words, the variable *d* is assumed endogenous, whereas exogeneity is required for causal inference using OLS and decomposition (I) [10].

Even though empirical work suggests that the socioeconomic variable is an important predictor for *health* (see, e.g., [2, 11, 13, 17, 18, 25]), the OLS regression-based methodology of decomposition (I) does not provide the right framework to use this result for the explanation of *socioeconomic inequality of health*. To bridge the gap between the empirical result and the regression-based decomposition methodology, we propose using a SEM approach (see below) that unifies these contrasting themes.

#### A rank-oriented decomposition

Erreygers and Kessels [4] introduced a rank-oriented decomposition that relies on a linear regression model for the fractional rank deviations. Assuming that the variables *z*_1_,*z*_2_,...,*z*_*q*_ are the relevant variables to explain the socioeconomic ranks, this model is given by

8 $$ d_{i}=\gamma_{0}+\gamma_{1}z_{1,i}+\gamma_{2}z_{2,i}+...+\gamma_{q}z_{q,i}+\xi_{i}  $$

where *ξ*_*i*_ is an error term. Substituting the right-hand side of this model for *d*_*i*_ in the ‘covariance definition’ of the *GC* in (5) and working out the result, we arrive at the rank-oriented decomposition, henceforth referred to as decomposition (II):

9 $$ GC=2\sum\limits_{g=1}^{q}\gamma_{g}Cov(h,z_{g})+2Cov(h,\xi)  $$

Decomposition (II) has a similar structure to decomposition (I) because it decomposes the Generalized Concentration Index into a sum of *q* explained contributions, with each of these equal to a covariance weighted by a regression coefficient, and a residual or unexplained component, which is also a covariance. In line with good practice to exclude the socioeconomic variable *d* from the OLS regression for *h* in decomposition (I), Erreygers and Kessels [4] also advise against the inclusion of *h* in the OLS regression for *d* because it would artificially result in a zero residual covariance in decomposition (II). In that case, the variable *h* is assumed endogenous. In order to make room for a possible effect of health on socioeconomic status in the framework of decomposition (II), we recommend using a SEM procedure that describes the feedback mechanism between these two variables (see below).

#### A two-dimensional simultaneous decomposition

To give proper attention to the bivariate nature of the Generalized Concentration Index, Erreygers and Kessels [4] proposed a set of two-dimensional decompositions that investigate the health levels *h* and the fractional rank deviations *d* simultaneously. The most salient of these decompositions is based on the bivariate multiple regression model that explains both variables simultaneously. It is typical of the bivariate multiple regression that a common set of *p* variables *s*_1_,*s*_2_,...,*s*_*p*_ is used to explain *h* and *d*. The bivariate multiple regression has the following form:

10 $$ h_{i} = \lambda_{0}+\lambda_{1}s_{1,i}+\lambda_{2}s_{2,i}+...+\lambda_{p}s_{p,i}+\psi_{i}  $$

11 $$ d_{i} = \pi_{0}+\pi_{1}s_{1,i}+\pi_{2}s_{2,i}+...+\pi_{p}s_{p,i}+\chi_{i}  $$

where *ψ*_*i*_ and *χ*_*i*_ are error terms. It is assumed that *μ*_*ψ*_=*μ*_*χ*_=0 and that the 2*p* covariances *C* *o* *v*(*s*_*j*_,*χ*) and *C* *o* *v*(*ψ*,*s*_*j*_) are zero.

Applying the ‘covariance definition’ of the *GC* in (5) to the bivariate multiple regression model leads to the simultaneous decomposition, henceforth referred to as decomposition (III):

$$  \begin{aligned} GC&=2\sum\limits_{j=1}^{p}\lambda_{j}\pi_{j}Var(s_{j})\,+\,2\sum\limits_{j=1}^{p}\sum\limits_{g=j+1}^{p}\!(\lambda_{j}\pi_{g}\,+\,\lambda_{g}\pi_{j})Cov(s_{j},s_{g}) \\ &\quad+2Cov(\psi,\chi)  \end{aligned}  $$

It consists of *p* single-variable terms *λ*_*j*_ *π*_*j*_ *V* *a* *r*(*s*_*j*_) which capture the direct effect of the *p* explanatory variables, $\frac {p(p-1)}{2}$ two-variable terms (*λ*_*j*_ *π*_*g*_+*λ*_*g*_ *π*_*j*_)*C* *o* *v*(*s*_*j*_,*s*_*g*_) which capture the correlation structure between the explanatory variables, and a residual component which is proportional to the covariance between the two error terms.

In the next section, we show that the simultaneous decomposition based on the bivariate multiple regression model is also the same decomposition that we obtain from applying a SEM regression approach.

### A flexible SEM approach for decomposition analysis

Perhaps the most pertinent critique of the bivariate multiple regression model as a basis for two-dimensional decomposition is the one that questions the assumption that the same set of *p* variables explains both the health variable *h* and the fractional rank deviation *d*. The challenge rests on the grounds that the determinants of health and socioeconomic status need not be the same. Moreover, the bivariate multiple regression model seems inflexible in the sense that it does not include *h* as a predictor in the equation for *d* and *d* as a predictor in the equation for *h*. Empirical evidence has shown, however, that health is largely influenced by socioeconomic status. It might also be the case that socioeconomic status is influenced by health, implying that both variables influence one another reciprocally [7].

To overcome the criticisms of the bivariate multiple regression model, we propose the specification of a structural or simultaneous equation model (see, e.g., [8]: chapter 10; [22]: chapter 5) which allows for different sets of predictors for *h* and *d* as well as the addition of *d* as a predictor in the equation for *h* and of *h* as a predictor in the equation for *d*. These structural equations are meant to represent causal relationships among the variables in the model.

We assume that the variables *x*_1_,*x*_2_,...,*x*_*k*_ with *x*_*k*_=*d* are the relevant variables in the equation for *h* and *z*_1_,*z*_2_,...,*z*_*q*_ with *z*_*q*_=*h* are the relevant variables in the equation for *d*. We then have the following structural model of two equations:

12 $$ h_{i}=\beta_{0}+\sum\limits_{j=1}^{k-1}\beta_{j}x_{j,i}+\beta_{k}d_{i}+\varepsilon_{i}   $$

13 $$ d_{i}=\gamma_{0}+\sum\limits_{g=1}^{q-1}\gamma_{g}z_{g,i}+\gamma_{q}h_{i}+\xi_{i}   $$

In this SEM, the variables *h* and *d* are assumed endogenous or jointly determined by the system of simultaneous equations. The random error terms *ε* and *ξ* affect both *h* and *d* (which is made clear by rewriting (13) in terms of *h*), suggesting a correlation between each of the endogenous variables and each of the random error terms. The remainder of the variables in the SEM are assumed exogenous or determined outside the system.

Because of the endogeneity of the variables *h* and *d*, OLS regression cannot be relied upon to produce consistent estimates of the parameters of the equations. Instead, a GMM estimation procedure using instrumental variable (IV) or two-stage least squares (2SLS) estimation is needed to consistently estimate all parameters of the SEM [9]. This requires the introduction of at least one instrumental variable or instrument for each equation. An instrument for an equation is strongly correlated with the right-hand side endogenous variable of that equation but uncorrelated with the equation’s error term. Moreover, an instrument does not have a direct effect on the response variable, and thus it does not belong on the right-hand side of the equation as an explanatory variable. It is therefore only a tool or instrument to solve the endogeneity problem, hence the name. Using an efficient GMM estimator, a necessary condition for identification of the two-equation SEM is that each equation has at least one exogenous variable that is not present in the other equation.

Once the SEM is estimated, Eq. (12) can be used as the input for decomposition (I) and Eq. (13) as the input for decomposition (II). In this way, by using an efficient GMM estimation procedure instead of OLS, the contribution of *d* in decomposition (I) and of *h* in decomposition (II) is duly measured.

Substituting the right-hand side of (13) for *d*_*i*_ in (12) and the right-hand side of (12) for *h*_*i*_ in (13), we obtain:

$${} h_{i}=\beta_{0}+\sum\limits_{j=1}^{k-1}\beta_{j}x_{j,i}+\beta_{k}\left[ \gamma_{0}+\sum\limits_{g=1}^{q-1}\gamma_{g}z_{g,i}+\gamma_{q}h_{i}+\xi_{i}\right] +\varepsilon_{i} $$

$${} d_{i}=\gamma_{0}+\sum\limits_{g=1}^{q-1}\gamma_{g}z_{g,i}+\gamma_{q}\left[ \beta_{0}+\sum\limits_{j=1}^{k-1}\beta_{j}x_{j,i}+\beta_{k}d_{i}+\varepsilon_{i} \right] +\xi_{i} $$

Rearranging terms and assuming that *β*_*k*_ *γ*_*q*_≠1, we arrive at the following reformulation of the model, which is called the reduced form of the SEM:

14 $$ \begin{aligned} h_{i}&=\frac{\beta_{0}+\beta_{k}\gamma_{0}}{1-\beta_{k}\gamma_{q}} +\sum\limits_{j=1}^{k-1}\frac{\beta_{j}}{1-\beta_{k}\gamma_{q}} x_{j,i}\\&\quad+\sum\limits_{g=1}^{q-1}\frac{\beta_{k}\gamma_{g}}{1-\beta_{k}\gamma_{q}} z_{g,i}\!+\frac{\varepsilon_{i}+\beta_{k}\xi_{i}}{1-\beta_{k}\gamma_{q}}  \end{aligned}  $$

15 $$ \begin{aligned} d_{i}&=\frac{\gamma_{0}+\beta_{0}\gamma_{q}}{1-\beta_{k}\gamma_{q}} +\sum\limits_{j=1}^{k-1}\frac{\beta_{j}\gamma_{q}}{1-\beta_{k}\gamma_{q}} x_{j,i}\\&\quad+\sum\limits_{g=1}^{q-1}\frac{\gamma_{g}}{1-\beta_{k}\gamma_{q}}z_{g,i}+ \frac{\xi_{i}+\gamma_{q}\varepsilon_{i}}{1-\beta_{k}\gamma_{q}}  \end{aligned}  $$

The reduced-form equations express each endogenous variable, *h* and *d*, in terms of the exogenous variables, $x_{1},x_{2},...,x_{k-1}\phantom {\dot {i}\!}$ and $z_{1},z_{2},...,z_{q-1}\phantom {\dot {i}\!}$, and the intercept, plus an error term. If variable $\phantom {\dot {i}\!}x_{j^{\ast }}$ is equal to variable $\phantom {\dot {i}\!}z_{g^{\ast }}$ – nothing excludes this case – then the coefficient of the variable in question in (14) will be $\phantom {\dot {i}\!}(\beta _{j^{\ast }}+\beta _{k}\gamma _{g^{\ast }})/(1-\beta _{k}\gamma _{q})$, and in (15) $\phantom {\dot {i}\!}(\beta _{j^{\ast }}\gamma _{q}+\gamma _{g^{\ast }})/(1-\beta _{k}\gamma _{q})$. The reduced-form equations describe the (equilibrium) impact after allowing for all interactions between the endogenous variables to work themselves out.

Like the bivariate multiple regression model (10)–(11), the reduced form of the SEM in (14)–(15) is characterized by the same set of explanatory variables, which we note as $s_{1}, s_{2},...,s_{p}\phantom {\dot {i}\!}$. Eqs. (14) and (15) can then be simplified as:

16 $$ h_{i}= \lambda_{0}+ \lambda_{1}s_{1,i}+ \lambda_{2}s_{2,i} +...+ \lambda_{p}s_{p,i} + \psi_{i}  $$

17 $$ d_{i}= \pi_{0}+ \pi_{1}s_{1,i}+ \pi_{2}s_{2,i} +...+ \pi_{p}s_{p,i} + \chi_{i}  $$

The parameters *λ*_0_,*λ*_1_,...,*λ*_*p*_ and *π*_0_,*π*_1_,...,*π*_*p*_ in (16)–(17) are called reduced-form parameters. The error terms *ψ*_*i*_ and *χ*_*i*_ are called reduced-form errors.

The reduced-form Eqs. (16)–(17) are equivalent to the bivariate multiple regression model (10)–(11), and can be consistently estimated by OLS since the right-hand side variables are exogenous and uncorrelated with the random errors *ψ*_*i*_ and *χ*_*i*_. This shows that, using a SEM regression approach, we end up with decomposition (III) based on the bivariate multiple regression model. Within the SEM framework, this decomposition incorporates the feedback mechanism between the variables *h* and *d*, which are allowed to depend on different sets of predictors. As a result, the above analysis answers to the criticisms of the bivariate multiple regression model and the resulting decomposition (III).

In our empirical study described in the next section, we show that a SEM regression analysis forms the basis for a proper use of decompositions (I), (II) and (III).

## Results

### Data description

For comparison the data are the same as those used by Erreygers and Kessels [4]. They come from the 2011 Demographic and Health Survey (DHS) of Ethiopia and are confined to children under the age of five.

The response variables in decompositions (I), (II) and (III) are the health variable *h* and the fractional rank deviation *d*. The health variable *h* is actually an ill-health variable: the degree of stunting or malnutrition. It is defined on the unit interval [ 0,1] and provides information on the depth of child malnutrition. It is measured using the child’s height-for-age standard deviation or *z*-score which is the difference between the height of a child and the median height of a child of the same age and sex in a well-nourished reference population, divided by the standard deviation in the reference population. The new WHO child growth population was chosen as the reference population. The degree of stunting is stated relative to the threshold of minus two standard deviations of the median of the reference population. Children with a *z*-score greater than this threshold are designated as not stunted and are assigned a zero degree value. The other children are stunted and are assigned a value in the unit interval that is proportional to the magnitude of their *z*-score, where a *z*-score of minus six standard deviations corresponds to the maximum value of one. In total, taking into account the sample weights provided by the DHS, 44*%* of the children in the dataset are stunted. The fractional rank deviation *d* was obtained by ranking the children’s households according to their wealth status using the wealth indices constructed by the DHS from a principal component analysis on all household living conditions and assets. In the computation sample weights were taken into account so that, in effect, the variable *d* stands for the weighted fractional rank deviation.

The set of explanatory variables is the same as the one used by Erreygers and Kessels [4] except for the variable ‘time to water source’, which turned out to be insignificant in their decomposition analyses. The variables are: age and sex of the child, education of the mother and her partner or husband, urban or rural residence, access to safe drinking water, and satisfactory sanitation. In addition to that, the child’s age is specified nonlinearly in the regression models using a squared term, which is mean-centered to remove multicollinearity with the linear term. Furthermore, safe drinking water and satisfactory sanitation are defined along the lines proposed by the WHO and UNICEF. ‘Safe drinking water’ includes the following sources of water supply: piped water (piped into dwelling, piped into yard or plot, or public tap), water from a protected well, tube well or borehole, water from a protected spring, and rainwater. ‘Satisfactory sanitation’ includes the following sanitation infrastructure: a flush toilet (flush to piped sewer system, septic tank or pit latrine), a pit latrine with slab, a ventilated improved pit (VIP) latrine and a composting toilet.

Table 1 shows a summary of all the variables with their descriptive statistics taking into account the sample weights. The data contain information on 9262 children under the age of five. The value for the *GC* equals −0.0136 using either the ‘product definition’ in (1) or the ‘covariance definition’ in (5). Its negative sign reveals higher rates of child malnutrition amongst the poor, that is, a socioeconomic inequality of malnutrition to the disadvantage of the poor. In the next two sections, we apply the various approaches described in this paper to compute decompositions (I), (II) and (III), which we express in percentages. We first discuss the decomposition results from using an OLS regression approach, and then those from using a SEM approach. We performed all regression analyses using the econometric software package EViews 9.

**Table 1:** Mean, standard deviation and description of all variables

Variable	Mean	SD	Description
Degree of stunting	0.1252	0.2073	Height-for-age *z*-score (WHO) scaled to the interval [0,1]
			Degree of stunting >0 if height-for-age *z*-score <−2 SD
Weighted fractional rank deviation	0	0.2952	Based on the wealth indices provided by the DHS
Age of child	29.8571	17.8084	In months
Squared age of child	303.3724	270.6317	Term is mean-centered: (age of child −29.8571)^2^
Sex of child	0.5140	0.5110	Male (1), female (0)
Residence type	0.1237	0.3366	Urban (1), rural (0)
Education of mother	1.3446	2.8587	In years
Education of partner/husband	2.7439	3.8141	In years
Safe drinking water	0.4614	0.5097	Available (1), not available (0)
Satisfactory sanitation	0.1234	0.3362	Available (1), not available (0)

### Decomposition results using OLS regression

#### Decompositions (I) and (II)

The results for decompositions (I) and (II) depend on the specification of the OLS regression model used. For decomposition (I), an important comparison to study is that between the exclusion and the inclusion of the weighted fractional rank deviation *d* in the regression for the degree of stunting *h*. For decomposition (II), we carry out a similar analysis, comparing the results from excluding and including *h* in the regression for *d*. Table 2 contains the coefficients for the two sets of regressions for *h* and *d* as well as the *t*- and *F*-statistics and significances. We corrected standard errors for heteroskedasticity by using White’s heteroskedasticity-consistent standard errors.

**Table 2:** OLS regressions for the degree of stunting *h* and the weighted fractional rank deviation *d*, where *d* has been excluded and included in the regression for *h* and *h* has been excluded and included in the regression for *d*

	*h*				*d*			
	Excluding *d*		Including *d*		Excluding *h*		Including *h*	
	Coefficient	*t*-stat	Coefficient	*t*-stat	Coefficient	*t*-stat	Coefficient	*t*-stat
Constant	0.1305	15.80 ^∗∗∗^	0.1212	14.16 ^∗∗∗^	-0.1720	-18.54 ^∗∗∗^	-0.1627	-16.99 ^∗∗∗^
Age of child	0.0016	10.94 ^∗∗∗^	0.0016	11.10 ^∗∗∗^	0.0003	1.82 ^◇^	0.0005	2.41 ^∗^
Squared age of child	-0.0001	-13.49 ^∗∗∗^	-0.0001	-13.49 ^∗∗∗^	0.0000	0.02	0.0000	-0.80
Sex of child	0.0135	2.30 ^∗^	0.0139	2.36 ^∗^	0.0065	0.98	0.0074	1.12
Residence type	-0.0255	-2.18 ^∗^	-0.0122	-1.02	0.2470	22.30 ^∗∗∗^	0.2452	21.84 ^∗∗∗^
Education of mother	-0.0036	-3.43 ^∗∗∗^	-0.0030	-2.87 ^∗∗^	0.0106	8.06 ^∗∗∗^	0.0103	7.87 ^∗∗∗^
Education of partner/husband	-0.0030	-3.31 ^∗∗∗^	-0.0022	-2.38 ^∗^	0.0146	13.49 ^∗∗∗^	0.0144	13.28 ^∗∗∗^
Safe drinking water	0.0033	0.53	0.0103	1.60	0.1289	18.12 ^∗∗∗^	0.1291	18.18 ^∗∗∗^
Satisfactory sanitation	-0.0170	-2.03 ^∗^	-0.0110	-1.28	0.1118	12.12 ^∗∗∗^	0.1106	11.96 ^∗∗∗^
*d*	−	−	-0.0539	-4.19 ^∗∗∗^	−	−	−	−
*h*	−	−	−	−	−	−	-0.0712	-4.17 ^∗∗∗^
*F*		96.55 ^∗∗∗^		90.11 ^∗∗∗^		765.62 ^∗∗∗^		687.07 ^∗∗∗^
*R* ^2^	0.0770		0.0806		0.3983		0.4006	
*N*	9262		9262		9262		9262	

^◇^ *p*<0.1, ^∗^ *p*<0.05, ^∗∗^ *p*<0.01, ^∗∗∗^ *p*<0.001

Using OLS regression, the *t*-statistics indicate that the variables *d* and *h* are highly significant in the regressions for *h* and *d*, respectively. In other words, *h* is very much influenced by *d*, and vice versa, *d* is very much influenced by *h*. Furthermore, the regression results for *h* are greatly affected when *d* is included as a regressor, whereas the regression results for *d* do not seem to differ much when *h* is included. When *d* is excluded in the regression for *h*, all variables are significant at the 5% level except for safe drinking water. However, when *d* is included in the regression for *h*, two more variables besides safe drinking water turn out to be insignificant, namely residence type and satisfactory sanitation. Also, education of the mother and her partner become less significant when including *d* as a regressor. Consequently, it seems that in the regression for *h*, some of the variation explained by these variables is being attributed to *d*. In contrast, when *h* is included in the regression for *d*, only the child’s age variable is affected in the sense that it becomes more significant. Although we argue that the OLS framework is not the right methodology to estimate the regression models with *d* and *h* as regressors, because of the correlated nature of the cross-sectional data, we suspect that the regression model for *h* including *d* makes more sense than the regression model for *d* including *h*.

**Fig. 1:**
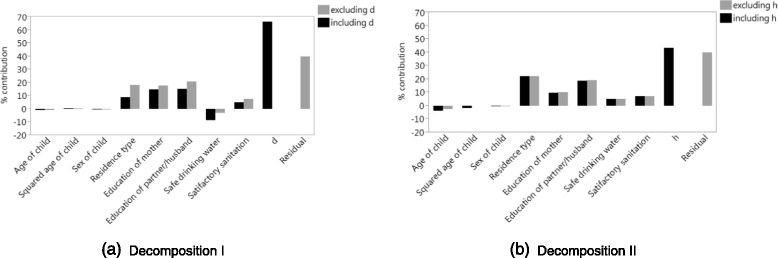
Percentage contributions from **a** decomposition (I), excluding and including *d*, and **b** decomposition (II), excluding and including *h*, using the OLS regressions from Table 2

Using the two regressions for *h*, excluding and including *d*, we computed two versions of decomposition (I), and using the two regressions for *d*, excluding and including *h*, we computed two versions of decomposition (II). The percentage contributions of these decompositions are shown in Table 3 and visualized in Fig. 1. An important observation is that decomposition (I) has a zero residual component when *d* is included and decomposition (II) has a zero residual component when *h* is included. Also, the contribution of *d* in decomposition (I) and of *h* in decomposition (II) are by far the largest, being 66.08% and 43.08%, respectively, and seem to capture all residual variation on top of their real contributions, compared to the large residual value of 39.76% in decomposition (I) excluding *d* and in decomposition (II) excluding *h*. As discussed previously, this result is an artefact of including either socioeconomic status or health as a variable in the decompositions that aim to explain the correlation between these variables.

**Table 3:** Percentage contributions from decomposition (I), excluding and including *d*, and decomposition (II), excluding and including *h*, using the OLS regressions from Table 2

	I		II	
	Excluding *d*	Including *d*	Excluding *h*	Including *h*
Age of child	-1.04	-1.05	-2.79	-3.72
Squared age of child	0.20	0.20	0.04	-1.55
Sex of child	-0.27	-0.28	-0.31	-0.36
Residence type	18.26	8.74	22.18	22.02
Education of mother	17.65	14.86	10.03	9.79
Education of partner/husband	20.60	15.18	18.88	18.61
Safe drinking water	-2.81	-8.66	4.91	4.91
Satisfactory sanitation	7.65	4.94	7.30	7.22
*d*	−	66.08	−	−
*h*	−	−	−	43.08
Residual	39.76	0	39.76	0
Total	100.00	100.00	100.00	100.00

One might thus inadvertently conclude that the contributions of *d* and *h* are very large in decompositions (I) and (II). However, for decomposition (I), the contribution of *d* exceeds the residual term from the same decomposition when *d* is excluded by a factor of 1.66, whereas for decomposition (II), the contribution of *h* is about the same as the residual term from the same decomposition excluding *h*. Compared to these residual terms, the contribution of the socioeconomic variable in decomposition (I) may be real and large, but not as large as 66.08%, whereas the contribution of the health variable in decomposition (II) may not be real. Also, similar to the regression results, when *d* is included in decomposition (I), the contributions of most other variables are smaller in absolute magnitude than when *d* is excluded. In contrast, when *h* is included in decomposition (II), the contributions of the other variables seem largely unaffected.

#### Decomposition (III)

**Fig. 2:**
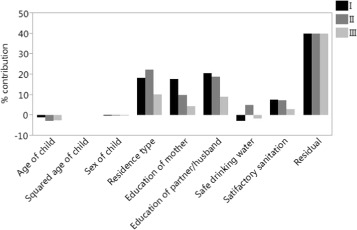
Percentage contributions from direct effects related to decompositions (I), (II) and (III) using the OLS regressions for *h* excluding *d* and for *d* excluding *h* from Table 2

We computed decomposition (III) starting from the bivariate multiple regression model, the coefficients of which are the same as those from the univariate regressions for *h* excluding *d* and for *d* excluding *h*, shown in Table 2. Table 4 contains the individual percentage contributions of decomposition (III). As indicated by Erreygers and Kessels [4], the column and row totals of the contributions of decomposition (III) relate to decompositions (I) and (II) from the regressions for *h* excluding *d* and for *d* excluding *h*. The contribution of the residual term in decomposition (III) is therefore the same as in decompositions (I) and (II), equating to 39.76%. Table 5 contains a summary presentation of decomposition (III) showing the direct and combined or correlated percentage contributions. Similar to the results of Erreygers and Kessels [4], the total of the combined or correlated contributions is almost twice as large as the total of the direct contributions. As a comparison, Fig. 2 contains the direct percentage contributions of decomposition (III) as well as the contributions from decomposition (I) excluding *d* and from decomposition (II) excluding *h*.

**Table 4:** Percentage contributions from decomposition (III) in relationship with decompositions (I) and (II) using the OLS regressions for *h* excluding *d* and for *d* excluding *h* from Table 2

	Age	Squared	Sex	Residence	Education	Education	Safe	Satisfactory		Total
	child	age child	child	type	mother	partner	water	sanitation	*χ*	(I)
Age child	-2.49	0.00	-0.02	0.16	0.59	0.96	-0.46	0.21	−	-1.04
Squared age child	-0.19	0.04	0.03	0.31	0.19	0.75	-0.88	-0.03	−	0.20
Sex child	-0.01	0.00	-0.32	0.03	0.02	0.00	0.04	-0.03	−	-0.27
Residence type	0.00	0.00	0.00	10.05	1.51	2.64	2.65	1.42	−	18.26
Education mother	-0.04	0.00	0.00	4.99	4.41	4.75	2.16	1.39	−	17.65
Education partner	-0.04	0.00	0.00	5.22	2.86	8.99	2.13	1.45	−	20.60
Safe water	0.00	0.00	0.00	-0.66	-0.16	-0.27	-1.57	-0.14	−	-2.81
Satisfactory sanitation	-0.01	0.00	0.00	2.09	0.62	1.08	0.83	3.03	−	7.65
*ψ*	−	−	−	−	−	−	−	−	39.76	39.76
Total (II)	-2.79	0.04	-0.31	22.18	10.03	18.88	4.91	7.30	39.76	100.00

**Table 5:** Percentage direct and combined contributions from decomposition (III) using the individual contributions from Table 4

	Direct effect	Combined effect						
		Age	Squared	Sex	Residence	Education	Education	Safe
		child	age child	child	type	mother	partner	water
Age child	-2.49							
Squared age child	0.04	-0.19						
Sex child	-0.32	-0.02	0.03					
Residence type	10.05	0.15	0.31	0.03				
Education mother	4.41	0.54	0.19	0.01	6.50			
Education partner	8.99	0.92	0.75	0.00	7.86	7.60		
Safe water	-1.57	-0.46	-0.88	0.04	1.99	2.00	1.86	
Satisfactory sanitation	3.03	0.21	-0.03	-0.03	3.51	2.01	2.52	0.69
Component total	22.13				38.11			
Residual	39.76							
Total	100.00							

Assuming that all explanatory variables in the bivariate multiple regression model are the exogenous variables in a two-equation SEM for the estimation of *h* and *d*, decomposition (III) takes into account the mutual dependency between *h* and *d* and thus captures the net or reduced effects of the explanatory variables upon both *h* and *d*. In the next section, we further discuss the relevant decompositions obtained by using a SEM approach.

### Decomposition results using a SEM approach

#### SEM estimation

A first step in a SEM regression analysis for the estimation of *h* and *d* as endogenous variables in Eqs. (12)–(13) is to define the exogenous variables for each equation as well as the instrumental variables for GMM analysis. Looking at the OLS regression results in Table 2, we learn that the variables ‘child’s age’, both its linear and squared term, and ‘sex of child’ are important predictors for *h*, but not for *d*, whereas the variables ‘residence type’, ‘safe drinking water’ and ‘satisfactory sanitation’ are important predictors for *d*, but not for *h*. We have therefore removed the variables ‘residence type’, ‘safe drinking water’ and ‘satisfactory sanitation’ from the equation for *h*, and used ‘residence type’ and ‘satisfactory sanitation’ as instruments for *d*. We did not include ‘safe drinking water’ as an instrument because we obtained a more powerful GMM analysis by not considering this variable. Similarly, we have removed the variables ‘child’s age’, both its linear and squared term, and ‘sex of child’ from the equation for *d*, and used all three terms as instruments for *h*. For each equation in the SEM, we then have at least one exogenous variable that is not present in the other equation, so that our system is identified.

We estimated the SEM in (12)–(13) using a feasible efficient two-step GMM procedure for robust covariance estimation in the presence of heteroskedasticity [26] using EViews 9. This procedure is also known as two-stage instrumental variables (2SIV) or heteroskedastic two-stage least squares (H2SLS). Table 6 contains the GMM regression coefficients for the two-equation SEM as well as the OLS regression coefficients, for comparison. Regarding the GMM analysis, Table 6 includes the *t*-, Hansen’s *J*- and Cragg-Donald *F*-statistics and significances. Hansen’s *J*-statistic has a *χ* ^2^-distribution under the null hypothesis that the instruments for an equation in the SEM are valid. The *J*-statistics for the two equations in the SEM are not significant at the 5% level so that we conclude that all our instruments are valid. The Cragg-Donald *F*-statistic is used to test for weak instruments or instruments that are not highly correlated with an equation’s right-hand side endogenous variable. The Cragg-Donald *F*-statistics for the two equations in the SEM are highly significant, meaning that the instruments for each equation are strong.

**Table 6:** GMM regressions (and OLS regressions for comparison) for the structural equation model (SEM) which includes the degree of stunting *h* and the weighted fractional rank deviation *d* as endogenous variables

	*h*				*d*			
	GMM		OLS		GMM		OLS	
	Coefficient	*t*-stat	Coefficient	*t*-stat	Coefficient	*t*-stat	Coefficient	*t*-stat
Constant	0.1187	13.52 ^∗∗∗^	0.1240	15.32 ^∗∗∗^	-0.1700	-16.01 ^∗∗∗^	-0.1493	-26.15 ^∗∗∗^
Age of child	0.0017	11.18 ^∗∗∗^	0.0016	11.13 ^∗∗∗^	−	−	−	−
Squared age of child	-0.0001	-13.48 ^∗∗∗^	-0.0001	-13.55 ^∗∗∗^	−	−	−	−
Sex of child	0.0143	2.41 ^∗^	0.0138	2.34 ^∗^	−	−	−	−
Residence type	−	−	−	−	0.2502	22.55 ^∗∗∗^	0.2457	21.94 ^∗∗∗^
Education of mother	-0.0022	-1.81 ^◇^	-0.0033	-3.36 ^∗∗∗^	0.0108	8.01 ^∗∗∗^	0.0102	7.80 ^∗∗∗^
Education of partner/husband	-0.0014	-1.27	-0.0024	-2.63 ^∗∗∗^	0.0148	13.37 ^∗∗∗^	0.0144	13.21 ^∗∗∗^
Safe drinking water	−	−	−	−	0.1288	17.96 ^∗∗∗^	0.1296	18.23 ^∗∗∗^
Satisfactory sanitation	−	−	−	−	0.1132	12.17 ^∗∗∗^	0.1108	11.97 ^∗∗∗^
*d*	-0.0987	-3.46 ^∗∗∗^	-0.0559	-4.67 ^∗∗∗^	−	−	−	−
*h*	−	−	−	−	0.0826	1.25	-0.0621	-3.73 ^∗∗∗^
*R* ^2^	0.0767		0.0796		0.3895		0.3996	
*N*	9262		9262		9262		9262	
*J*		0.42		−		2.69		−
Cragg-Donald *F*		917.43 ^∗∗∗^		−		194.31 ^∗∗∗^		−

^◇^ *p*<0.1, ^∗^ *p*<0.05, ^∗∗^ *p*<0.01, ^∗∗∗^ *p*<0.001

Using a GMM regression, the *t*-statistics indicate that the health variable *h* is largely influenced by the weighted fractional rank deviation *d*, but there is no feedback or two-way influence in the sense that the weighted fractional rank deviation *d* is not affected by *h*. This result is different from the result from the OLS regression shown in Table 6 where *h* is highly significant in the regression for *d* (see also the discussion above). Furthermore, most exogenous variables in the GMM analysis of the SEM are significant at the 5% level, except in the GMM regression for *h*, where ‘education of the mother’s partner’ is insignificant and ‘education of the mother’ is only significant at the 10% level.

#### Decompositions

**Fig. 3:**
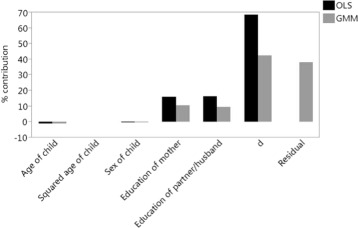
Percentage contributions from decomposition (I) using the SEM equation for *h* and the GMM and OLS regression coefficients from Table 6

Because the GMM analysis has shown that *d* has a significant impact on *h*, but not vice versa, we use the GMM regression for *h* from Table 6 as our input for decomposition (I) and we can simply use the OLS regression for *d* excluding *h*, from Table 2, as our input for decomposition (II). We refer to the section where we computed decomposition (II) based on this OLS regression. Note that whether or not we include the variables ‘child’s age’, both its linear and squared term, and ‘sex of child’ in the OLS regression for *d* does not make much difference in decomposition (II). Table 7 shows the percentage contributions of decomposition (I) based on the SEM equation for *h* in (12). We report the contributions using the GMM regression coefficients from Table 6 as well as the OLS regression coefficients for comparison. Figure 3 visualizes the two sets of contributions from decomposition (I). Note that, to compute the contribution of the weighted fractional rank deviation *d* using the GMM regression coefficients, we did not include *d* itself, but the *predicted* value of *d* resulting from the OLS regression of *d* on all the exogenous and instrumental variables in the SEM equation for *h*.

**Table 7:** Percentage contributions from decomposition (I) using the SEM equation for *h* and the GMM and OLS regression coefficients from Table 6

	GMM	OLS
Age of child	-1.06	-1.06
Squared age of child	0.20	0.20
Sex of child	-0.29	-0.28
Residence type	−	−
Education of mother	10.75	16.21
Education of partner/husband	9.67	16.47
Safe drinking water	−	−
Satisfactory sanitation	−	−
*d*	42.62	68.45
*h*	−	−
Residual	38.11	0
Total	100.00	100.00

Table 7 and Fig. 3 show that decomposition (I) using GMM regression has a large residual component of 38.11%, which is of the same size as that of decomposition (I) excluding *d* and using OLS regression. Furthermore, the contribution of *d* is much lower and more realistic using GMM instead of OLS. It was reduced from 68.45% using OLS to 42.62% using GMM, which is, however, still a substantial percentage. Also, the contributions of the variables ‘education of the mother’ and ‘education of the mother’s partner’ were lowered to a similar extent, approximately by a factor of 0.6, by using GMM instead of OLS.

Lastly, regarding decomposition (III), whether we use the bivariate multiple regression model or the SEM regression approach, we end up with the same decomposition (III) which we discussed previously.

## Discussion

Results reveal that the SEM for the estimation of health and socioeconomic status can easily be transformed into a bivariate multiple regression model for these variables, which, in the SEM framework, is also called the reduced form of the SEM. The SEM’s exogenous variables are the explanatory variables in each equation of this model format. We can then simply apply OLS to estimate the bivariate multiple regression model and use the regression coefficients as input for the two-dimensional simultaneous decomposition introduced by Erreygers and Kessels [4]. As such, we have shown that this decomposition takes into account the mutual dependency between health and socioeconomic status and captures the reduced effects of the explanatory variables upon health and socioeconomic status.

In the case that one wishes to use one of the main one-dimensional decompositions, the health-oriented decomposition proposed by Wagstaff et al. [25] or the rank-oriented decomposition (without a constant term) proposed by Erreygers and Kessels [4], a GMM analysis of the SEM is required for a proper application of these decompositions. However, if the GMM regressions indicate that socioeconomic status is insignificant in the equation for health or health in the equation for socioeconomic status, we can resort again to an OLS regression analysis of the particular equation(s) after having removed the insignificant endogenous variable(s). We would advise very strongly against applying OLS to the initial SEM equations.

The data used in our empirical study on child malnutrition in Ethiopia has been sufficiently rich to specify and estimate a SEM. The GMM analysis of the SEM confirms previous findings that health is largely influenced by socioeconomic status, but the opposite relationship appears not to hold. In the GMM analysis the effect of socioeconomic status on health is, however, indirect and measured by the instrumental variables ‘residence type’ and ‘satisfactory sanitation’. We used the GMM regression coefficients of the health equation in the health-oriented decomposition and the OLS regression coefficients of the socioeconomic status equation (without an explanatory health variable) in the rank-oriented decomposition. We recommend such modeling practice when computing one-dimensional decompositions. The contribution of socioeconomic status in the health-oriented decomposition turns out to be 42.62%, which is substantial and by far the largest. This contribution is, however, indirect and measured by the variables ‘residence type’ and ‘satisfactory sanitation’. The residual term is not zero, as when using OLS regression coefficients, but amounts to 38.11%, which is about the same size as the residual term from decompositions based on OLS regressions without health and socioeconomic status as explanatory variables.

Furthermore, we computed the two-dimensional simultaneous decomposition based on the bivariate multiple regression model, since this model is equivalent to the reduced form of the SEM. The total of the combined or correlated contributions in this decomposition is almost twice as large as the total of the direct contributions, and the residual term amounts to 39.76%. All in all, we can conclude that the SEM provides a flexible modeling framework for correctly applying the one- and two-dimensional decompositions and we therefore recommend it as a starting basis for decomposition analysis. Strictly speaking, only inequality indices with weighting functions that are independent of the predictors of health can be decomposed correctly [10].

For reasons of comparison, the empirical study presenting the SEM contains the same set of explanatory variables as the one used by Erreygers and Kessels [4]. Because this set is rather limited, the study should be seen as a pilot that can naturally be extended with more variables that may deepen the understanding of the determinants of child malnutrition in Ethiopia. Also, if data for different years were available, lagged versions of the variables could be incorporated in the SEM to explore changes in inequalities over different years. Such an approach should however be carefully compared to the Oaxaca-type decomposition technique [14] that has been especially developed for this purpose.

We emphasize that although the SEM of the empirical study has been corroborated by the data, this does not mean that it has been proven true. It just has not been falsified, but there may be competing models that would not have been falsified either. Also, the causal relationships implied by the SEM may be true, but strictly speaking, because of the non-experimental or correlational nature of the data, there is nothing in the SEM that magically transforms correlational data into causal conclusions. The same holds for OLS regression equations.

Finally, the SEM proposed in this paper is an observed-variables SEM because the endogenous variables health and socioeconomic status are observed or measured. A potentially interesting topic for further research would be to construct a SEM where the endogenous variables are not observed, but latent [12]. We refer to [15] for an application for measuring the outcome of Worksite Health Promotion Programs. Using a latent-variables SEM, the socioeconomic variable, when assumed latent, can be made directly dependent on a series of wealth-related variables. Also, instead of transforming the socioeconomic levels into ranks, another avenue would be to use the socioeconomic levels as they are, and to construct a level-dependent index of socioeconomic inequality of health, as proposed by Erreygers and Kessels [5], to which a SEM regression-based decomposition analysis can easily be applied.

## Conclusion

Empirical research has provided evidence that socioeconomic status, represented by a wealth- or income-related variable, is an important determinant of health. Vice versa, it is likely that health is an important determinant of socioeconomic status. However, to explain socioeconomic inequality of health, or the *correlation* between health and socioeconomic status, neither of the variables, health or socioeconomic status, can be used as an explanatory variable in an OLS regression-based decomposition approach, because we would then explain the bivariate dependent variable by one of its univariate components, which is meaningless. To unify the potentially bidirectional relationship between health and socioeconomic status with the regression-based decomposition methodology, we recommend using a structural or simultaneous equation model (SEM) which captures the feedback mechanism between health and socioeconomic status using a system of equations for these variables, which are assumed endogenous. More specifically, this two-equation model allows the inclusion of socioeconomic status as an explanatory variable for health and health as an explanatory variable for socioeconomic status, while providing consistent estimates using a two-step GMM estimation procedure. It also allows for the specification of different sets of determinants of health and socioeconomic status.
